# Alterations in hepatic mitotic and cell cycle transcriptional networks during the metabolic switch in broiler chicks

**DOI:** 10.3389/fphys.2022.1020870

**Published:** 2022-10-24

**Authors:** Julie A. Hicks, Brandon E. Pike, Hsiao-Ching Liu

**Affiliations:** Department of Animal Science, North Carolina State University, Raleigh, NC, United States

**Keywords:** broiler, metabolic switch, liver, RNA-seq, mitosis

## Abstract

During embryonic life, chicks mainly derive energy from hepatic oxidation of yolk lipids. After hatch, chicks must rely on carbohydrate-rich feed to obtain energy. This requires an abrupt and intensive switch of metabolic processes, particularly in the liver. We recently identified a number of transcriptional and post-transcriptional regulatory networks that work concordantly to tune metabolic processes during the metabolic switch. Here, we used delayed feeding post-hatch (48 h) to impede the metabolic switch in broilers. We used RNA-seq to identify hepatic transcriptome differences between late stage embryos (E18) and two-day-old chicks (D2), which were either fed-from-hatch (FED) or not fed (DLY). Between FED and E18, 2,430 genes were differentially expressed (fold-change≥ 2; FDR *p*-value 0.05), of these 1,237 were downregulated in FED birds and 1,193 were upregulated. Between DLY and E18, 1979 genes were differentially expressed, of these 1,043 were downregulated and 936 were upregulated in DLY birds. Between DLY and FED, 880 genes were differentially expressed, of these 543 were downregulated and 337 were upregulated in DLY birds. We found that in addition to disturbances in a number of metabolic pathways, unfed chicks had a widespread suppression of gene networks associated with cell proliferation, cell cycle progression and mitosis. Expression patterns suggest that hepatocytes of delayed-fed birds have abnormal mitosis and increased polyploidization. This suggests that post-hatch feed consumption maintains the rate and integrity of liver growth immediately, which in turn, likely helps facilitate the appropriate programming of hepatic metabolic networks.

## 1 Introduction

Chickens and birds in general, undergo a rapid and intensive metabolic shift during the embryonic-to-hatch transition. During the embryonic state, the chick mainly derives its energy from beta-oxidation of yolk lipids. After hatching, the chick’s main source of energy is derived from the conversion of feed-based carbohydrates into fatty acids (i.e. lipogenesis) (Reviewed by Noble and Cocchi, 1990). Similar to humans, this process occurs in the liver. In the embryonic state, yolk lipids are taken up into circulation *via* endocytosis. During the later stages of embryonic development and first days post-hatch yolk lipids are also transported to the intestines *via* the yolk stalk. This transport leads to high amounts of cholesteryl esters in the liver. Additionally, both increased glucose production and increased breakdown of hepatic glycogen stores leads to a rapid rise in glucose levels post-hatch (Noble and Cocchi, 1990). Chickens are naturally insulin resistant and easily tolerate glucose levels that would be fatal in mammals (Wang et al., 2017).

The hepatic molecular metabolic response to post-hatch feeding has been shown to include an increase in expression for genes involved in lipolysis and glycolysis and lower expression for genes involved in lipogenesis and cholesterol synthesis (Latour et al., 1995; Hicks et al., 2019). We and others have shown delaying feed intake for 48 h post-hatch impedes the initiation of lipogenic gene expression and can have long-term negative consequences on growth (Bhanja et al., 2009; Willemsen et al., 2010; Hicks et al., 2019). Comparison of the hepatic transcriptomes of E18 embryos to D3 chicks found over 800 differentially expressed transcripts, and particularly those involved in lipid and cholesterol synthesis (Hicks et al., 2017). Additionally, by means of RT-qPCR, we found that delaying access to feed for 48 h post-hatch alters the hepatic expression of a number of these genes (Hicks et al., 2019). For example, several genes involved in cholesterol synthesis, including MSMO1 and HMGCR were found to be significantly down-regulated in delayed fed chicks. Studies by us and others have shown that late stage embryos have high levels of ketone bodies and that these levels drop quickly upon feed consumption (Beis, 1985; Hicks et al., 2019). Ketone body levels in delayed-fed birds remain high until these birds are fed. Ketone body production is the result of increased fatty acid oxidation, typically due to periods of fasting or low dietary glucose intake, which generates acetyl-CoA at rates too high to be entirely processed by the TCA cycle (Reviewed by Watanabe et al., 2020). Ketone bodies released from the liver can then be used by other organs and tissues as an alternative energy source by reconversion into acetyl-CoA. In mammals, ketone bodies also function as triggers of the oxidative stress response (Nagao et al., 2016). Part of this response is the induction of the transcriptional regulator FOXO3, which in turn represses the expression of class I histone deacetylases, as part of a protective mechanism from oxidative stress (Nagao et al., 2016). We have previously shown that a similar mechanism is likely triggered during the metabolic switch in chickens, which may serve to protect the liver from oxidative damage due to excessive fatty acid oxidation (Hicks et al., 2017; Hicks et al., 2019). We also found that this process, at least partially, may be regulated by microRNA (Hicks et al., 2017; Hicks et al., 2019).

The vertebrate liver is one of the few tissues in which cells may naturally be polyploidy (containing more than two complete sets of homologous chromosomes) and/or multinuclear in a healthy individual (Kreutz et al., 2017). Under normal conditions, hepatocyte polyploidization has been linked to aging and hepatic injury (e.g., resections). Metabolic diseases such as steatosis and obesity can also lead to increased hepatocyte polyploidization (Singh et al., 2013). Though it has not been as intensively studied in poultry, several studies have found similar mechanisms of polyploidization as found in mammals. For example, an increase in hepatocyte polyploidization in chickens was shown to occur due to both age and liver injury, similar to mammals (Dzhivanyan and Ter-Oganyan, 1983).

In recent years there has been accumulating evidence of diverse and complex interactions between metabolic and cell cycle networks. For example, depletion of ACLY, the enzyme responsible for acetyl-CoA synthesis, reduces the acetylation of histones, which is required for DNA replication and cell cycle progression (Kaplon et al., 2015). Mice with high fat diet-induced steatosis display both reduced hepatocyte proliferation and a lag in DNA replication following liver resection (Caldez et al., 2020). Cyclins, along with cyclin-dependent kinases (CDKs) and CDK inhibitors (CKIs), coordinate the proper progression through the cell cycle (Johnson and Walker, 1999). Cyclins have been demonstrated to affect numerous metabolic pathways, including glycolysis and lipogenesis, *via* direct interactions with their major components, including FASN and ACACA (Hanse et al., 2012). Cyclins have also been shown to regulate mitochondria biogenesis and function (Hanse et al., 2012). Metabolic perturbations can have negative consequences on cell cycle progression (Leal-Esteban and Fajas, 2020). Cholesterol is an essential component of cell membranes and is thus required for cell proliferation (Fernández et al., 2004). Perturbations in both cholesterol synthesis and catabolism can lead to cell cycle arrest and abnormal mitosis. Cholesterol-starved cells have both reduced level of CDK1, a Ser/Thr protein kinase involved in the modulation of cell cycle progression and the centrosome cycle, and reduced rates of mitosis (Fernández et al., 2004).

We found that transcript levels of many hepatic metabolic genes that are altered in delayed-fed birds recover quickly after feeding, often reaching the comparable levels to fed-from-hatch chicks within 24–48 h (Hicks et al., 2019). However, as delayed feeding of newly hatched birds can cause long-term consequences on growth; our observation suggests that the rapid establishment of the molecular metabolic program post-hatch is vital for proper growth and development. To further explore the potential molecular networks associated with the metabolic switch, in this study we used RNA-seq to generate unbiased global transcriptional networks in delayed-fed broilers. We found that in addition to disruption of metabolic networks, there was also significant perturbations in the expression of mitotic and cell cycle regulators. This suggests that part of the metabolic switch entails the maintenance of the proper rate of liver growth, which in turn, programs the long-term metabolic state.

## 2 Materials and methods

### 2.1 Birds

All animal procedures were described previously (Hicks et al., 2019) and approved by North Carolina State University’s IACUC (protocol#17-179-A). Fertilized Ross 708 eggs were obtained from Perdue Farms Hatchery (Hurlock, MD, United States) and incubated under standard conditions. On embryonic day (E)18, eggs were weighed and eggs near the mean (57 g ± 2) were transferred to a hatchery. Birds hatching between 6:00 and 12:00 on E21/day of hatch (D)0 were feather sexed and weighed. Males weighing between 46 g and 48 g were randomly divided into two groups (*n* = 6). The first group (FED) received chick starter feed (Southern States, Richmond, VA, United States) *ad libitum* on D0. The second group (DLY) was not provided with feed. All birds were allowed *ad libitum* access to water from D0. On E18 and D2, birds were weighed, and plasma and liver samples were collected from six males in each group (E18, FED and DLY). Sex was confirmed by gonad identification. For embryonic birds the entire left lobe of the liver was snap frozen, for post-hatch birds approximately 1 G of the left lobe was snap frozen. All liver samples were stored at -80 °C until analyzed. Physiological measurements, including weight, Free Fatty Acids, β-hydroxybutyrate (Ketone Body), triglyceride, and glucose levels, were taken to confirm metabolic status and were described previously (Hicks et al., 2019).

### 2.2 RNA-seq library construction and sequencing

Total RNA was isolated from 50 mg of liver tissue using Tri-Reagent (Sigma-Aldrich St. Louis, MO, United States) following the manufacturer’s instructions and DNase-treated using a TURBO-DNA free kit (Thermo Fisher Scientific, Waltham, MA, United States) following the manufacturer’s instructions. RNA quality was assessed using an Agilent Technologies 2,100 Bioanalyzer with a high sensitivity RNA chip. All RNA samples had RIN values of 9.90–10. RNA-seq (mRNA) libraries were generated from 1 µg of total RNA per sample, using a NEBNext Poly(A) mRNA Magnetic Isolation Module (New England Biolabs), a NEBNext Ultra II RNA Library Prep kit for Illumina (New England Biolabs) and NEBNext Multiplex Oligos for Illumina (New England Biolabs) following the manufacturer’s instructions. Library quality was accessed using an Agilent Technologies 2200 TapeStation with D1000 tape. Libraries were quantified by RT-qPCR using a NGS Library Quantification kit (Takara Bio) following the manufacturer’s instructions. Libraries were diluted to 10nM, pooled and submitted to NCSU Genomic Sciences Laboratory for 75-bp single-end sequencing on a NextSeq 500 v2 flow cell (Illumina). FASTQ files have been submitted to the NIH SRA database: BioProject PRJNA723740, accession numbers SRR14298486-SRR14298503 (https://www.ncbi.nlm.nih.gov/bioproject/PRJNA723740). All sequencing data processing and analyses were performed using CLC Genomics Workbench (Qiagen). First, low quality reads (Phred<20) and any residual adaptor sequences were removed using the “Trim Reads” tool, the number of high quality reads per sample ranged from ∼27–34 million. Next read mapping and read count normalization were performed using the “RNA-seq Analysis” tool. Reads were mapped to the Gallus gallus genome (5.0) using the “Genome annotated with genes and transcripts” setting. Pairwise differential gene expression analyses were performed using RPKM values and the “Differential Expression in Two Groups” tool, which uses multi-factorial statistics based on a negative binomial Generalized Linear Model (GLM). These pairwise comparisons include both developmental timing and/or feeding status. The E18 vs. FED provides a developmental timing comparison under typical rearing conditions. The E18 vs. DLY comparison provides both a developmental timing and feeding status comparison. The FED vs. DLY comparison provides a feeding status comparison. Ingenuity Pathway Analysis (Qiagen) was then performed on the differential expression data using the integrated plugin.

## 3 Results

### 3.1 RNA-seq data statistics

**FIGURE 1:**
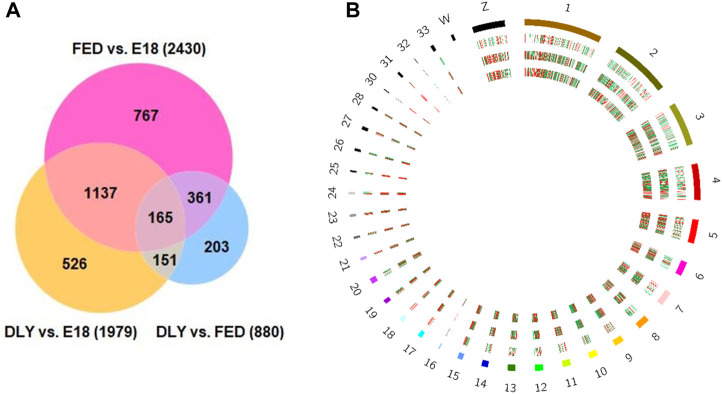
Number and genomic distribution of differentially expressed genes between E18 embryos and D2 fed-from-hatch (FED) chicks, E18 and D2 delayed-fed chicks (DLY) and between DLY and FED. **(A)** Venn Diagram of the number of differentially expressed genes (fold-change≥2; FDR *p*-value≤0.05) between E18 embryos and D2 fed-from-hatch (FED) chicks, E18 and D2 delayed-fed chicks (DLY) and between DLY and FED. **(B)** Circos plot of genomic distribution of differentially expressed genes. Red indicates upregulated genes and green indicates downregulated genes. Outermost ring is the Gallus gallus karyotype, followed the DLY vs. FED comparison, the DLY vs. E18 comparison and the innermost ring is the FED vs. E18 comparison. Cicros plot was generated using the Circos tool from galaxy (usegalaxy.org).

**FIGURE 2:**
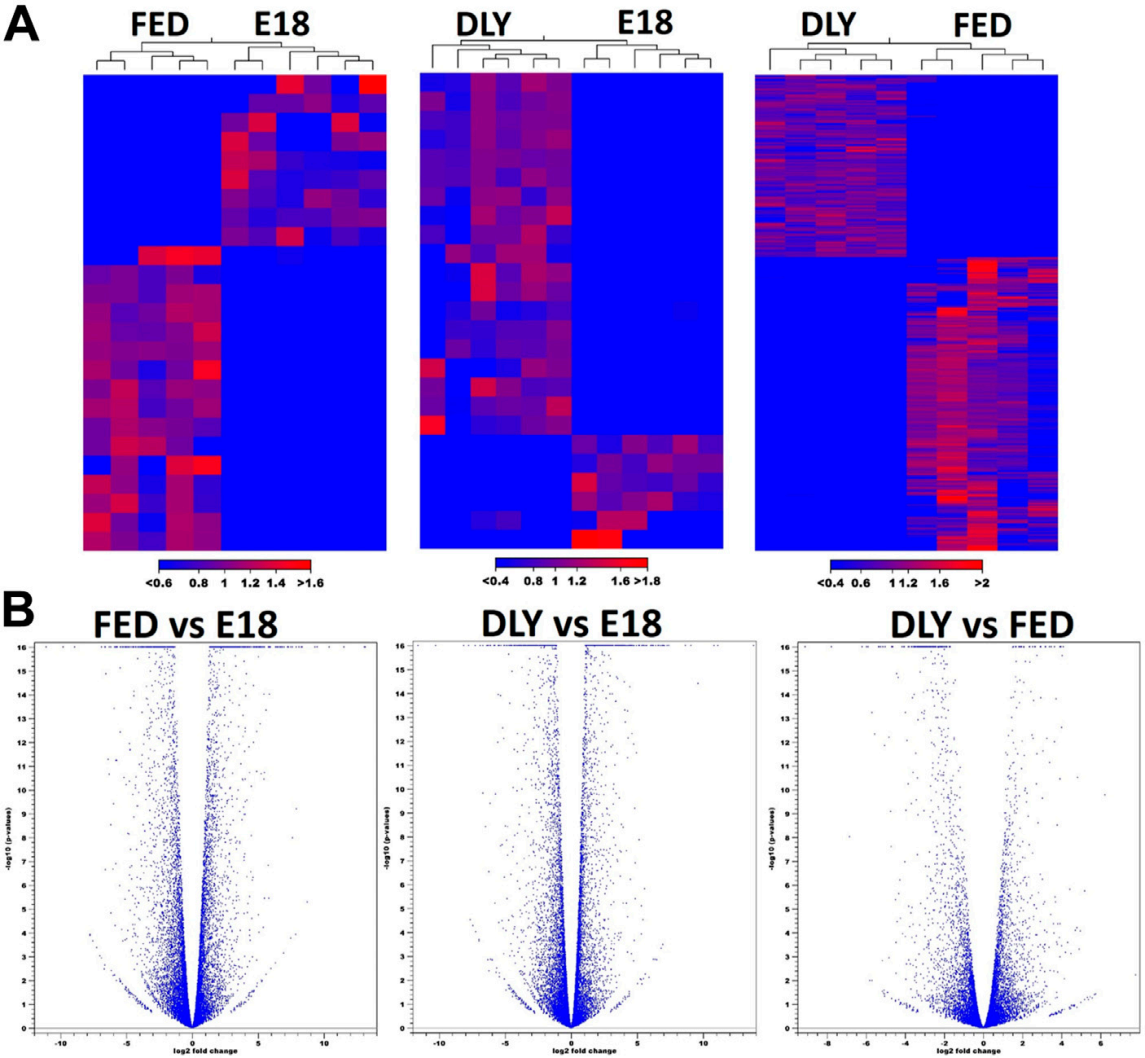
Heatmap anlaysis and volcano plot distributions of differentially expressed genes between E18 embryos and D2 fed-from-hatch (FED) chicks, E18 and D2 delayed-fed chicks (DLY) and between DLY and FED. **(A)** Heatmap analysis of differentially expressed genes (fold-change≥2; FDR *p*-value≤0.05) between E18 embryos and D2 fed-from-hatch (FED) chicks, E18 and D2 delayed-fed chicks (DLY) and between DLY and FED. Expression values used are normalized log CPM values. Red indicates higher expression and blue indicates lower expression. **(B)**. Volcano plots of FED vs. E18, DLY vs. E18, and DLY vs. FED pairwise comparisons. (fold-change≥2; FDR *p*-value≤0.05).

The number of reads passing quality filters ranged from 27–34 million. Of these, the percentage of reads mapped to the Gallusgallus genome ranged from 95.76% to 97.29%. Of the mapped reads, the reads mapping to protein-coding genes ranged from 97.57% to 98.80%. Expression values for all mapped genes are provided in Supplemental Table S1. The highest expressed gene in all groups was albumin (*ALB*). Between D2 fed-from-hatch chicks (FED) and E18 embryos (E18), 2,430 genes were differentially expressed (fold-change (FC)≥ 2; FDR *p*-value ≤0.05) (Figure 1A), of these, 1,237 were downregulated in FED birds and 1,193 were upregulated. Of these 2,430 genes, 773 genes had a 5-fold or greater difference in expression (410 downregulated; 363 upregulated). Between D2 delayed-fed chicks (DLY) and E18, 1979 genes were differentially expressed (FC ≥ 2; FDR *p*-value ≤0.05) (Figure 1A), of these 1,043 downregulated and 936 were upregulated in DLY birds. Out of the 1979 differentially expressed genes, 565 had a fold change ≥5, with 289 downregulated and 276 upregulated. Between DLY chicks and FED chicks 880 genes were differentially expressed (FC ≥ 2; FDR *p*-value 0.05) (Figure 1A), of these 543 were downregulated and 337 were upregulated in DLY birds. A total of 296 of these genes had a fold change of 5-fold or greater. Of note, out of these 296 genes, 234 were downregulated and only 62 were upregulated. Genomic distribution of differentially expressed genes for each pairwise comparison is shown in Figure 1B. Heatmap analysis, revealed distinct expression patterns for each group (Figure 2A). Volcano plots for each pairwise comparison are shown in Figure 2B.

### 3.2 Changes in the hepatic transcriptome between E18 embryos and D2 FED chicks

**FIGURE 3:**
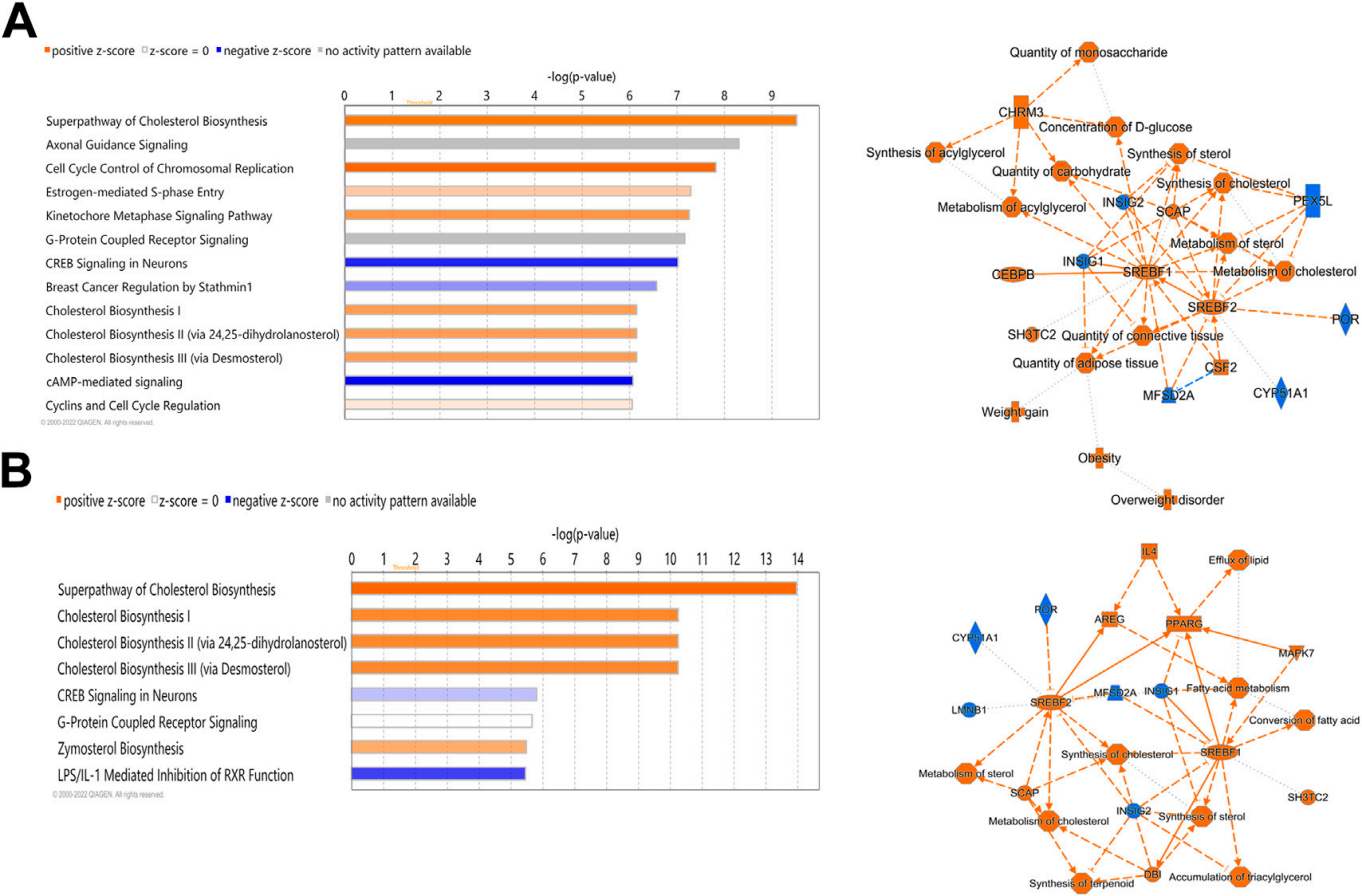
Canonical pathways of differentially expressed hepatic genes between FED chicks and E18 embryos. **(A)** Genes with a fold-change ≥2; FDR *p*-value≤0.05 and **(B)** Genes with a fold-change ≥5; FDR *p*-value≤0.05. Orange indicates a positive z-score; expression patterns of members predicts activation of the pathway. Blue indicates a negative z-score; expression patterns of members predicts inhibition of the pathway.

**FIGURE 4:**
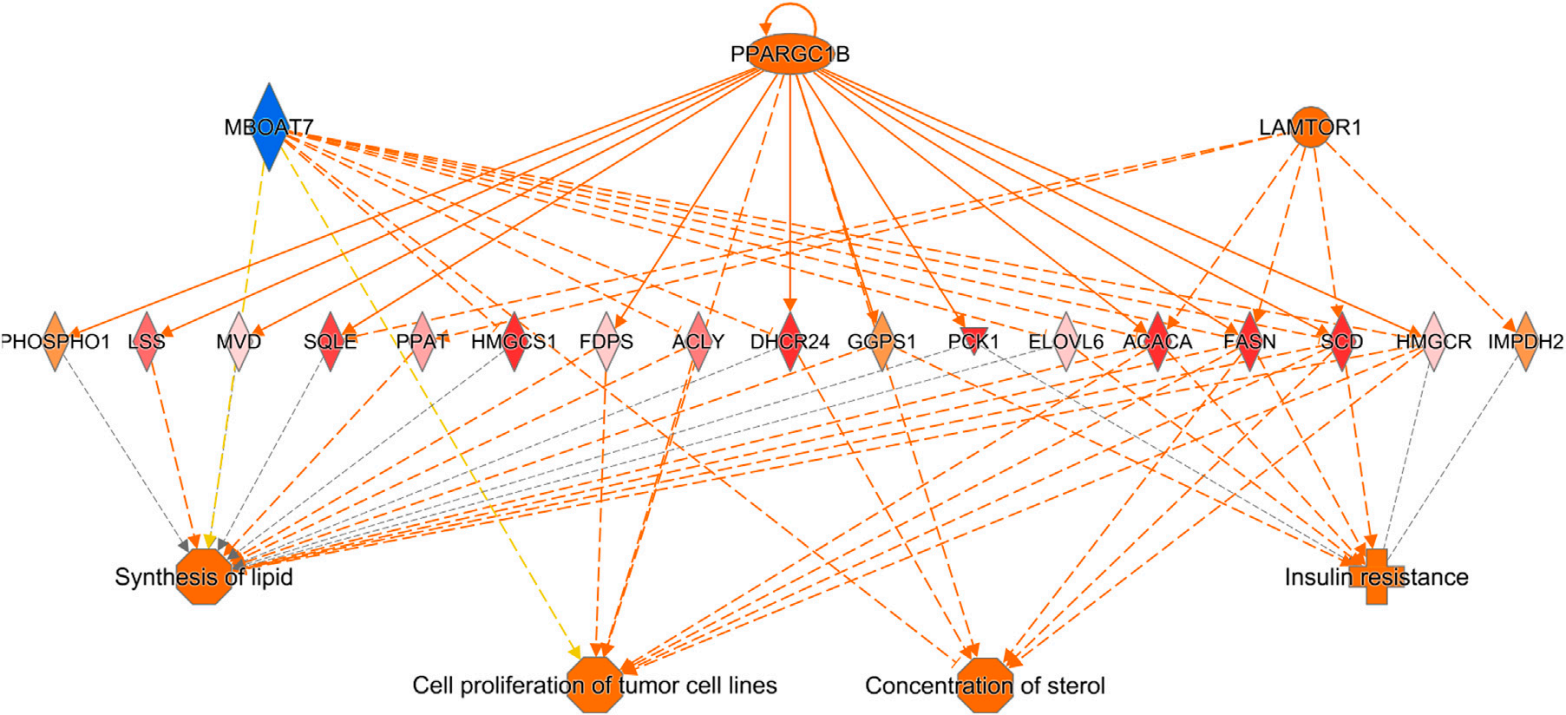
Network mapping of differentially expressed genes associated with cholesterol and fatty acid synthesis between FED chicks and E18 embryos. Network mapping of differentially expressed genes (fold-change≥5; FDR *p*-value≤0.05) associated with cholesterol and fatty acid synthesis between FED and E18. Many of the major enzymes associated with cholesterol and fatty acid synthesis were significantly upregulated in FED chicks relative to embryos. Red molecules were significantly upregulated and green molecules significantly downregulated in FED chicks. Orange indicates predicted activation and blue indicates predicted inhibition.

**FIGURE 5:**
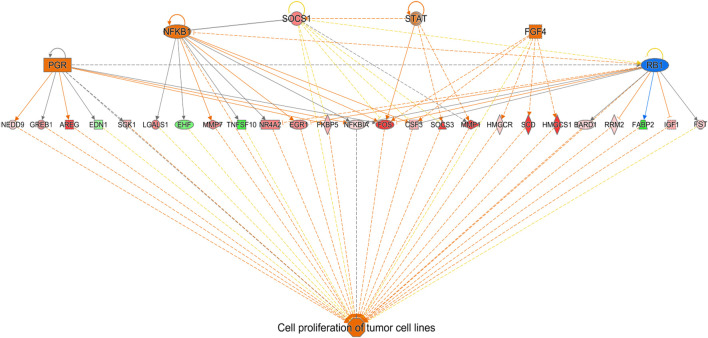
Network mapping of differentially expressed genes associated with cell proliferation between FED chicks and E18 embryos. Network mapping of differentially expressed genes (fold-change≥5; FDR *p*-value≤0.05) associated with cell proliferation between FED and E18. Genes associated with increased cell proliferation were also highly altered between FED and E18. Red molecules were significantly upregulated and green molecules significantly downregulated in FED chicks. Orange indicates predicted activation and blue indicates predicted inhibition.

Ingenuity Pathway Analysis (IPA) of differentially expressed hepatic genes (FC ≥ 2; FDR *p*-value ≤0.05) between E18 broilers and FED chicks, identified changes in the hepatic transcriptome after hatch are associated with the activation of both cholesterol biosynthesis pathways and pathways regulating the cell cycle and mitosis (Figure 3A). IPA analysis of genes with a greater than 5-fold difference between embryos and FED chicks indicated that the genes with the highest changes in hepatic expression after hatch are mainly involved in regulating cholesterol biosynthesis and fatty acid metabolism (Figure 3B). For example, many of the genes encoding enzymes involved in cholesterol and fatty acid synthesis were significantly upregulated in post-hatch chicks (Figure 4), including: *SQLE* (23.24-fold; *p* = 8.29E-29), *HMGCS1* (194.70-fold; *p* = 1.26E-211), *SCD* (643.08-fold; *p* = 1.93E-105), *ACACA* (26.12-fold; *p* = 1.44E-82), and *ACLY* (16.59-fold; *p* = 2.25E-76). Other genes with some of the largest differences between embryos and FED chicks include: *MMF1* (32.08-fold; *p* = 1.73E-05), *AREG* (33.59-fold; *p* = 4.41E-27), *EGR1* (15.90-fold; *p* = 2.32E-12), *FOS* (114.32-fold; *p* = 4.33E-33), *CSF3* (10.10-fold; *p* = 1.55E-05) and *SOCS3* (29.85-fold; *p* = 2.54E-20), which are involved in regulating cell proliferation (Figure 5).

### 3.3 Hepatic transcriptome differences between E18 embryos and D2 DLY chicks

**FIGURE 6:**
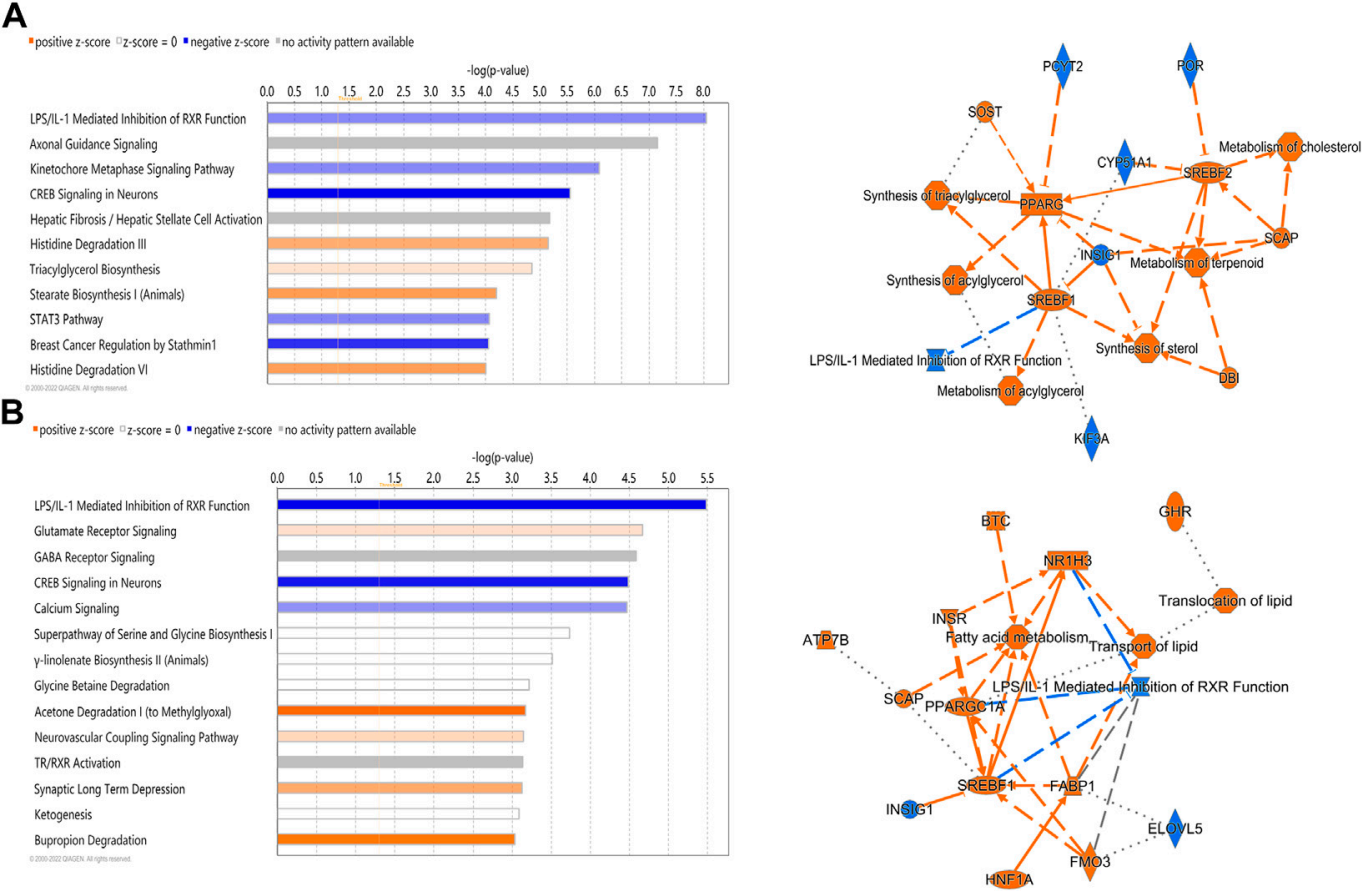
Canonical pathways of differentially expressed hepatic genes between DLY chicks and E18 embryos. **(A)** Genes with a fold-change ≥2; FDR *p*-value≤0.05 and **(B)** Genes with a fold-change ≥5; FDR *p*-value≤0.05. Orange indicates a positive z-score; expression patterns of members predicts activation of the pathway. Blue indicates a negative z-score; expression patterns of members predicts inhibition of the pathway.

**FIGURE 7:**
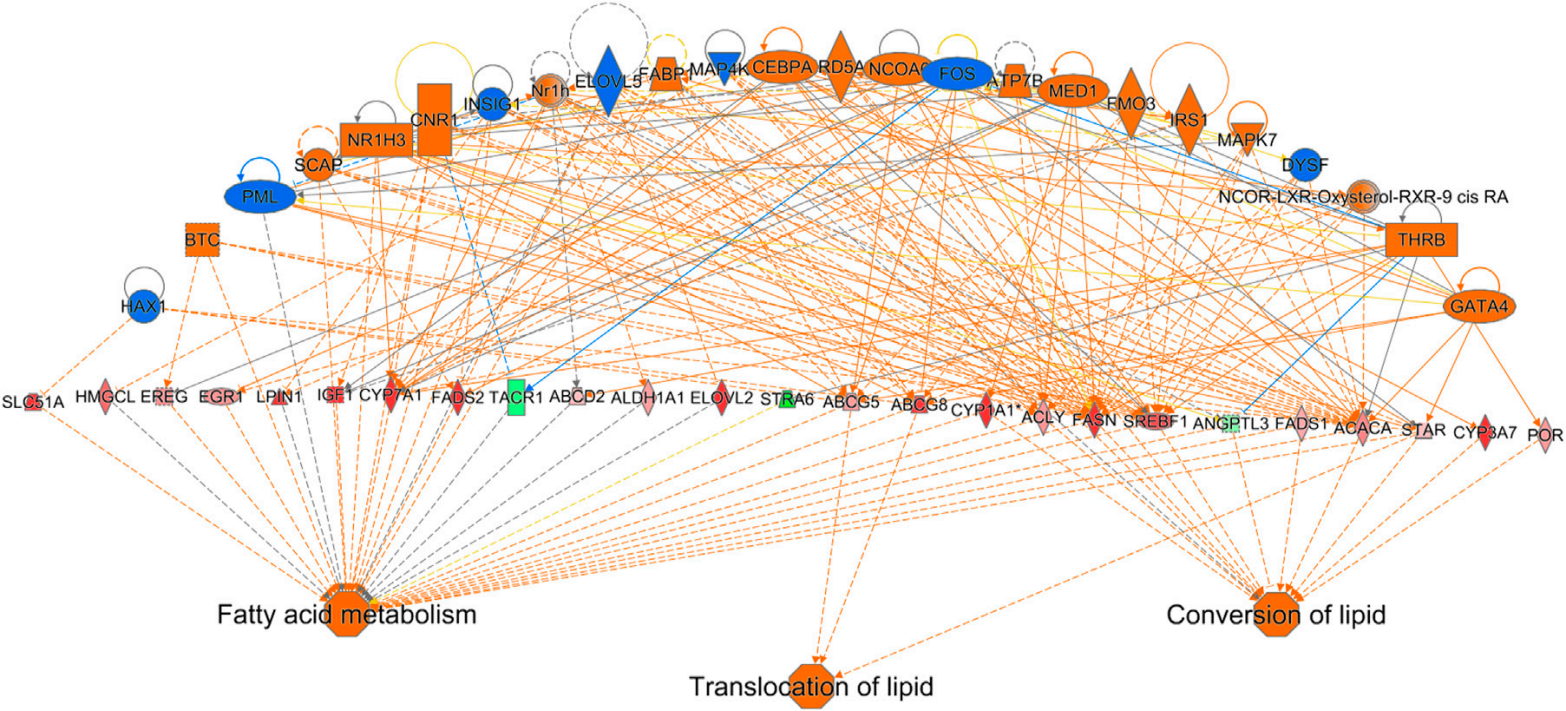
Network mapping of differentially expressed genes associated with fatty acid metabolism and lipid transport between DLY chicks and E18 embryos. Network mapping of differentially expressed genes (fold-change≥5; FDR *p*-value≤0.05) associated with fatty acid metabolism and lipid transport between DLY chicks and E18 embryos. Gene expression patterns suggest the biggest transcriptome differences between these two groups are involve genes associated with lipid transport and function. Red molecules were significantly upregulated and green molecules significantly downregulated in DLY chicks. Orange indicates predicted activation and blue indicates predicted inhibition.

**FIGURE 8:**
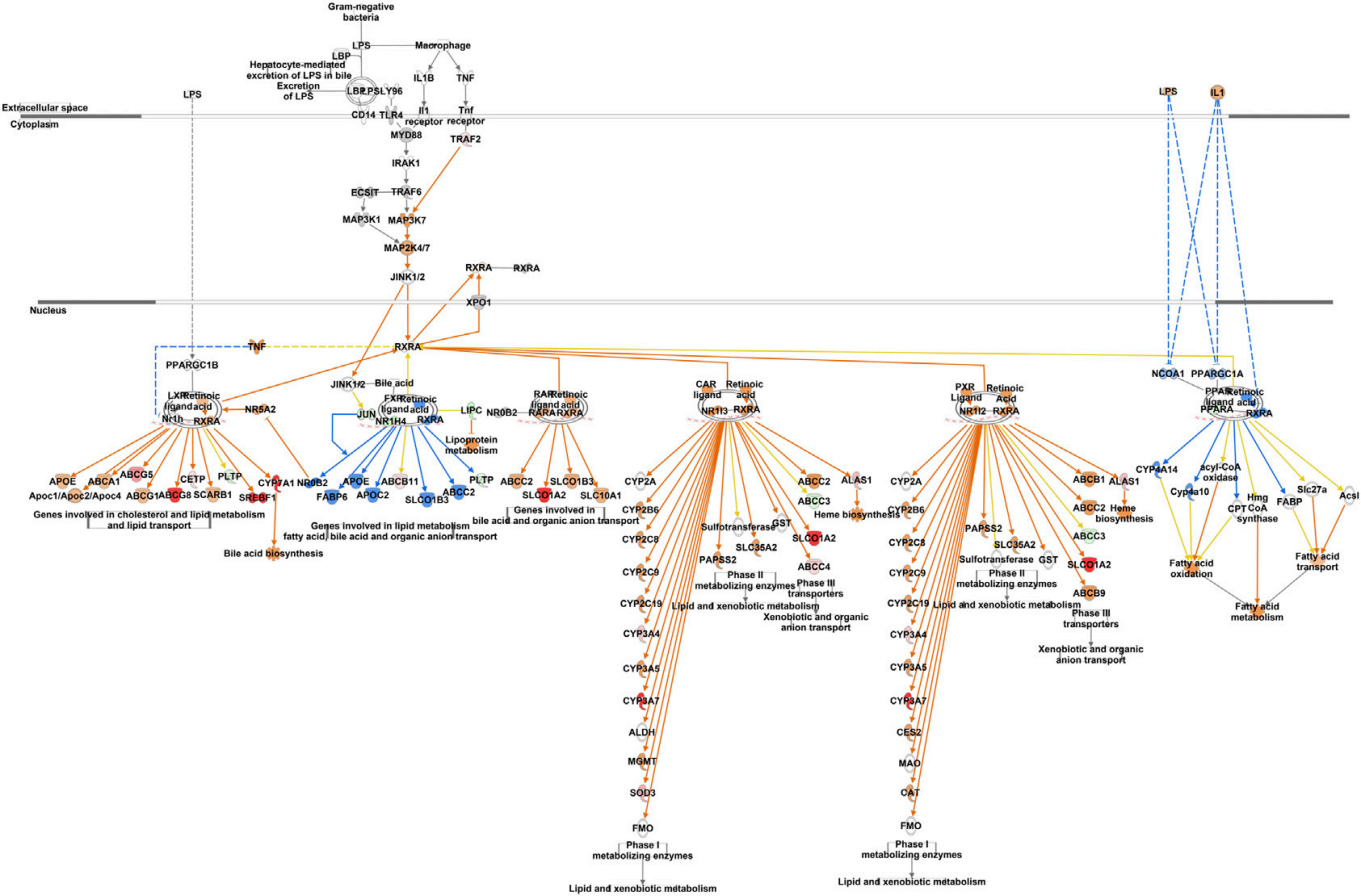
Network mapping of differentially expressed genes associated with RXR function between DLY chicks and E18 embryos. Network mapping of differentially expressed genes (fold-change≥5; FDR *p*-value≤0.05) associated with RXR function between DLY chicks and E18 embryos. Red molecules were significantly upregulated and green molecules significantly downregulated in DLY chicks. Orange indicates predicted activation and blue indicates predicted inhibition.

IPA analysis of differentially expressed hepatic genes (FC ≥ 2; FDR *p*-value ≤0.05) between E18 and DLY chicks indicates these genes are involved in the inactivation of LPS/IL-1 mediated Inhibition of RXR function, inactivation of Kinetochore Metaphase signaling, the activation of Histidine degradation and activation of metabolism of cholesterol (Figure 6A). IPA analysis of genes with a greater than 5-fold difference in expression between embryos and DLY chicks identified genes associated with lipid transport and Fatty acid metabolism, as well as those involved in Glutamate Receptor Signaling and Calcium Signaling (Figure 6B). Genes encoding several of the major members of fatty acid metabolic pathways and lipid transport pathways had much higher expression in the livers of DLY chicks compared to E18 embryos (Figure 7). These include, *SREBF1* (15.14-fold; *p* = 1.01E-94), *FADS2* (18.23-fold; *p* = 4.86E-20), *ELOVL2* (101.65; *p* = 4.09E-82), *IGF1* (27.01-fold; *p* = 2.14E-04), *SLC O 1A2* (397.56-fold; *p* = 3.63E-292) and *CYP7A1* (14609.65-fold; *p* = 4.86E-20). Several genes involved in RXR function (Figure 8) including, *ALAS1* (5.16-fold; *p* = 2.16E-18), *CYP3A7* (41.83-fold; *p* = 2.15E-127), and *CDO1* (2,204.95-fold; *p* = 3.17E-143), were also higher expressed in the DLY hepatic transcriptome compared to embryos.

### 3.4 Hepatic transcriptome differences between D2 broilers fed-from-hatch and delayed-fed

**FIGURE 9:**
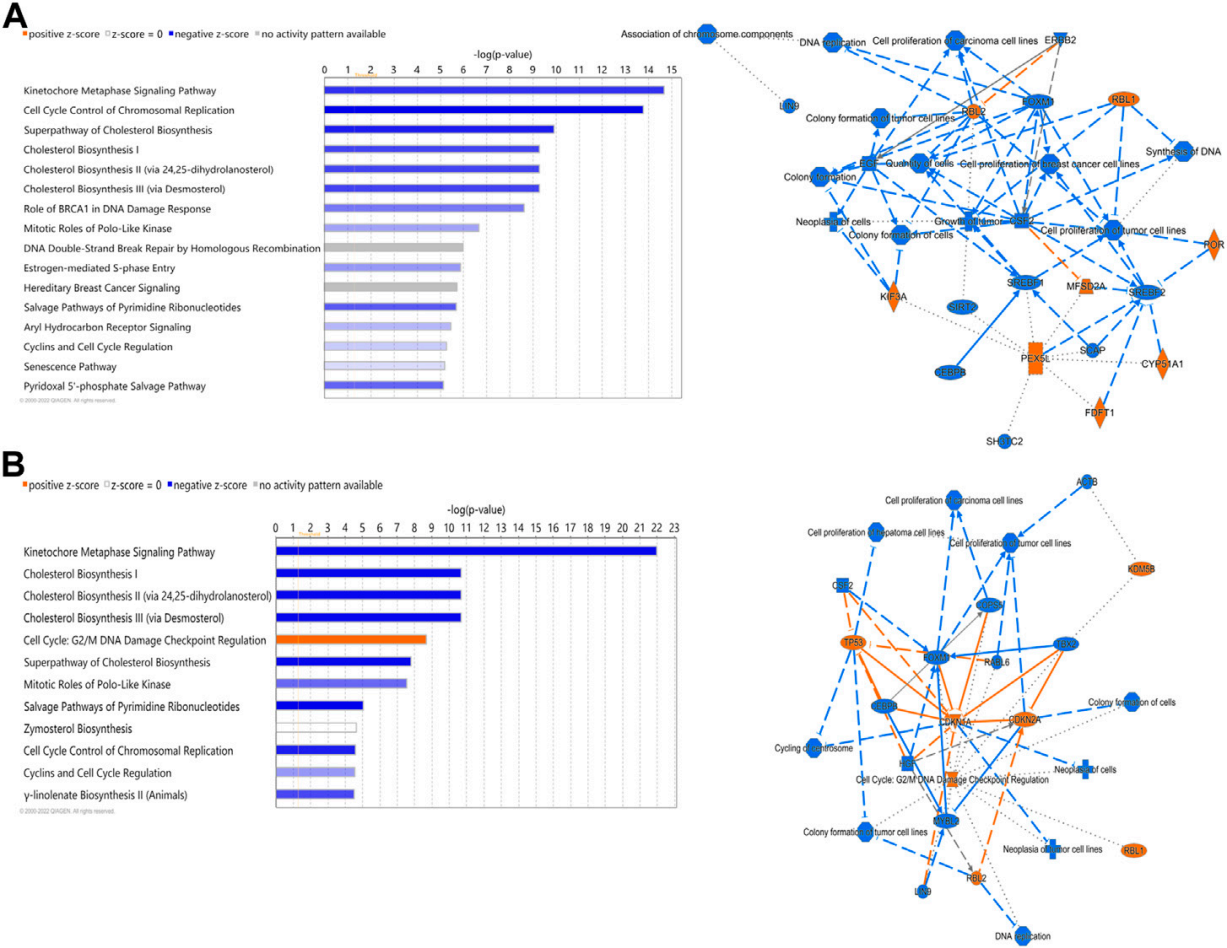
Canonical pathways of differentially expressed hepatic genes between DLY and FED chicks. **(A)** Genes with a fold-change ≥2; FDR *p*-value≤0.05 and **(B)** Genes with a fold-change ≥5; FDR *p*-value≤0.05. Orange indicates a positive z-score; expression patterns of members predicts activation of the pathway. Blue indicates a negative z-score; expression patterns of members predicts inhibition of the pathway.

**FIGURE 10:**
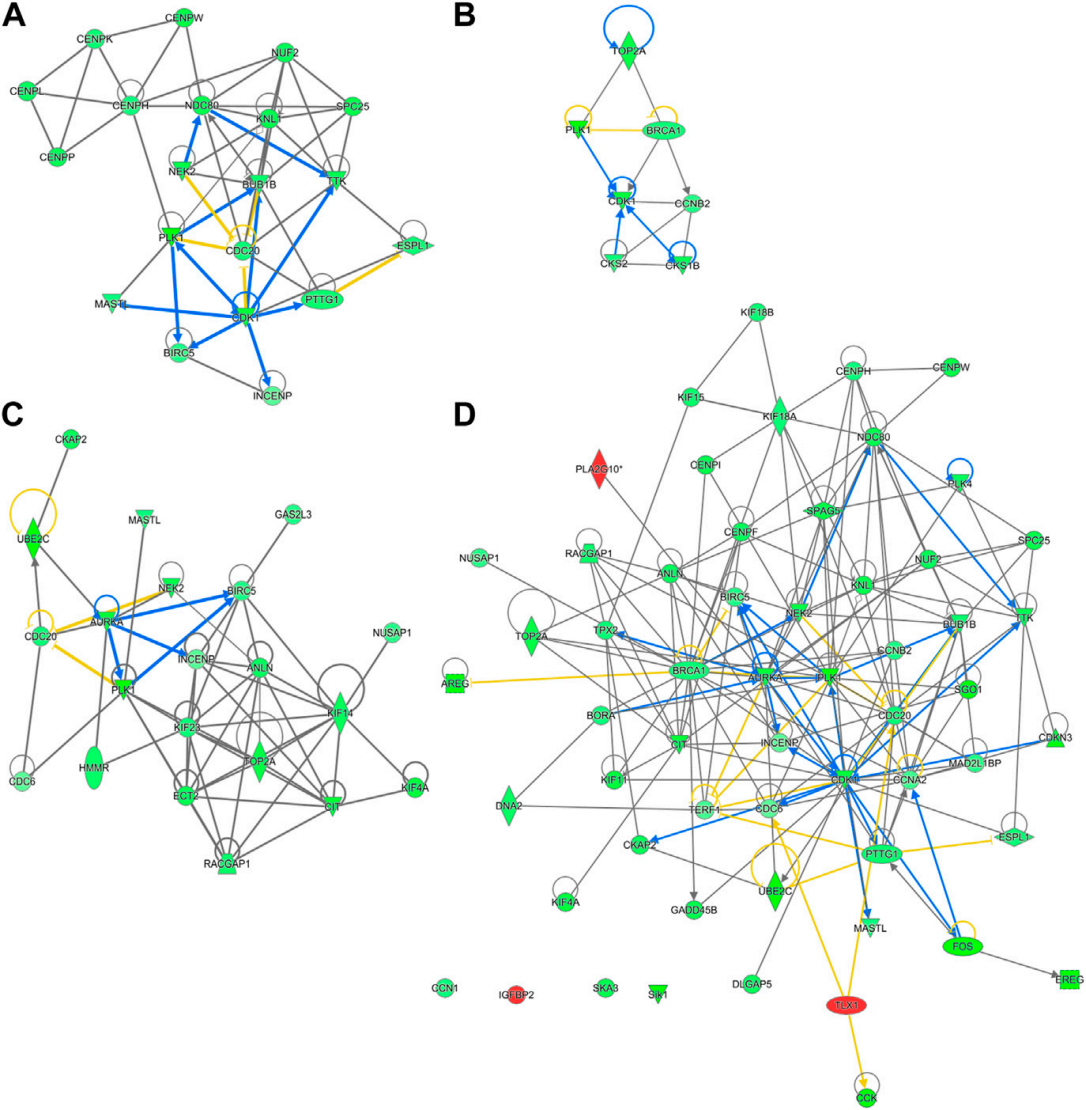
Network mapping of differentially expressed genes between DLY and FED chicks. Many genes associated with cell cycle progression and mitosis are altered (fold-change≥5; FDR *p*-value≤0.05) in DLY chicks compared to their FED counterparts, including those involved in **(A)** cytokinesis, **(B)** G2/M DNA Damage Checkpoint regulation, **(C)** kinetochore metaphase signaling, and **(D)** mitosis. Red molecules were significantly upregulated and green molecules significantly downregulated in DLY chicks. Orange indicates predicted activation and blue indicates predicted inhibition.

**FIGURE 11:**
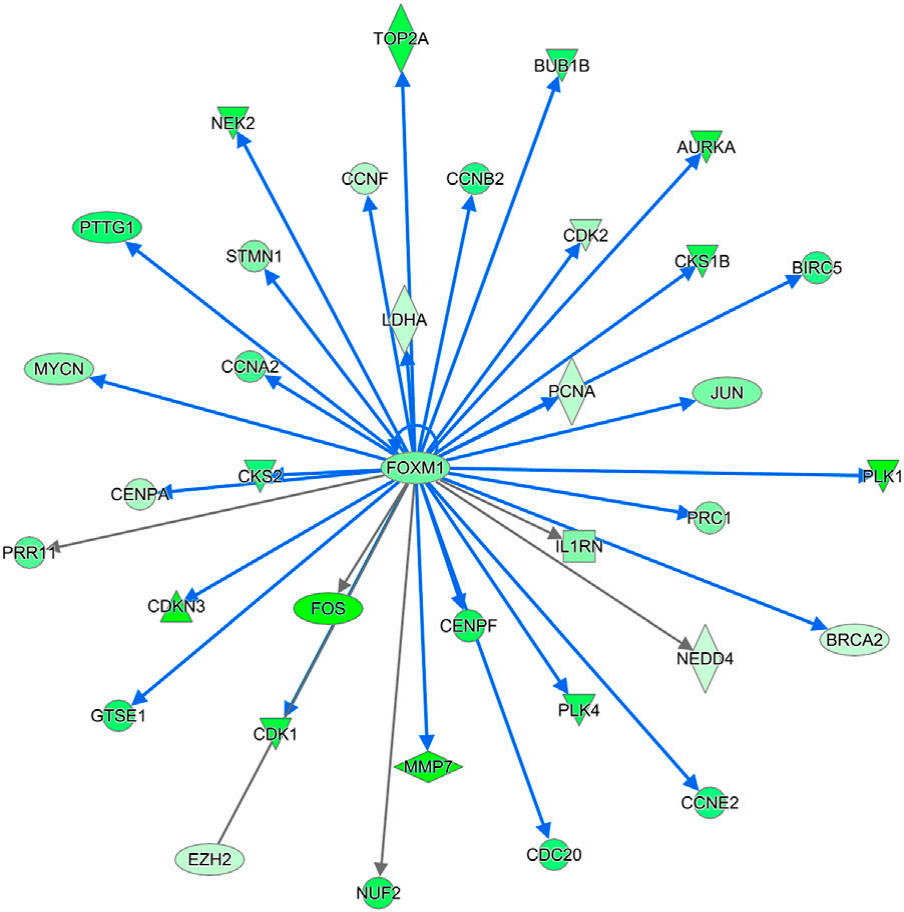
THE FOXM1 regulatory network. FOXM1 was significantly downregulated in DLY chicks relative to FED chicks. In turn many of the genes it regulates are also downregulated. FOXM1 is a master regulator of many cell proliferation processes. Red molecules were significantly upregulated and green molecules significantly downregulated in DLY chicks.

**FIGURE 12:**
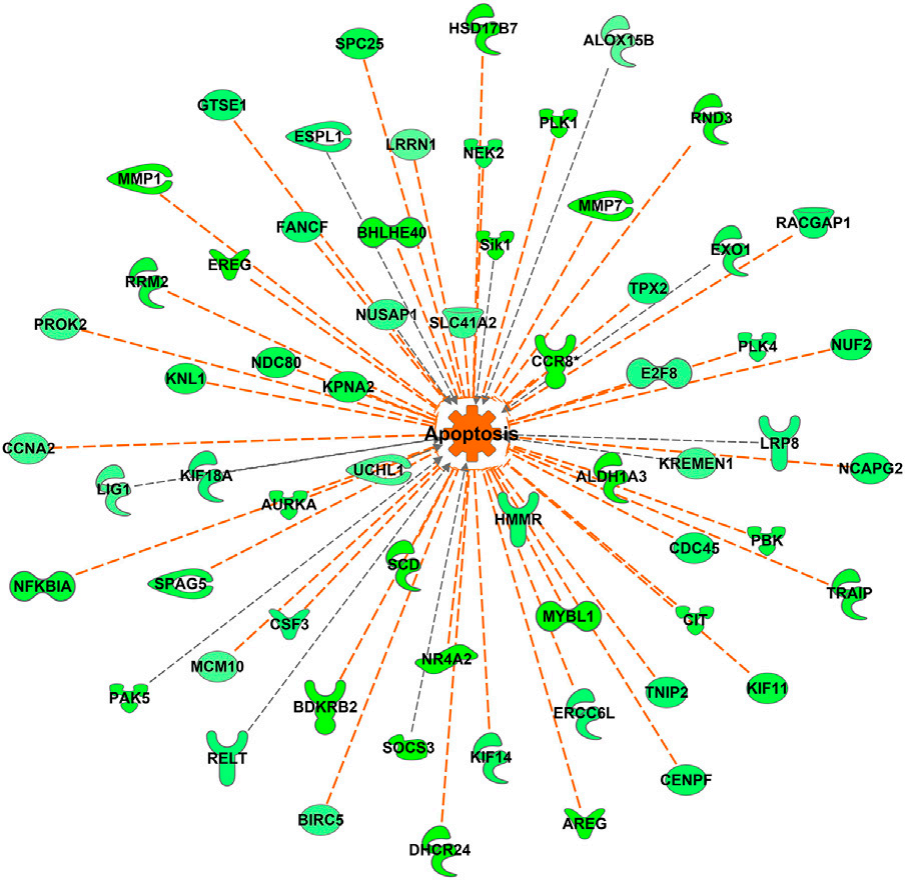
Apoptosis-associated genes differentially expressed between DLY and FED chicks. Many genes associated with apoptosis and cell viability were differentially expressed (fold-change≥5; FDR *p*-value≤0.05) between DLY and FED chicks. Expression patterns suggest there is increased apoptosis in the livers of DLY chicks compared to FED chicks. Green molecules significantly downregulated in DLY chicks. Orange indicates predicted activation.

Out of the 880 differently expressed (FC ≥ 2; FDR *p*-value ≤0.05) between D2 delayed-fed chicks and fed-from hatch chicks 543 (67%) were downregulated. This downregulation is even more pronounced in genes with a greater than 5-fold difference, with 79% (234 genes out of 296) downregulated in DLY birds. This suggests there is a general decrease in hepatic transcription processes in DLY chicks compared to their fed counterparts. IPA analysis of differentially expressed hepatic genes (FC ≥ 2; FDR *p*-value ≤0.05) between these groups found affected biological processes include, DNA replication, cell proliferation, Kinetochore Metaphase Signaling and Cell Cycle Control of Chromosomal Replication (Figure 9A). IPA analysis of genes with a greater than 5-fold difference in expression between these chicks found that some of the most affected processes include inactivation of Kinetochore Metaphase Signaling, inactivation of Mitotic Roles of Polo-like kinase, inactivation of cycling of centrosomes, inactivation of cholesterol biosynthesis, and activation of G2/M DNA Damage Checkpoint regulation (Figure 9B). The most upregulated gene in DLY birds was *ACOT12* (74.29-fold; *p* = 1.68E-10) and the most downregulated gene was *SCD* (−553.86-fold; *p* = 1.11E-90). Genes involved in cytokinesis (Figure 10A) significantly downregulated in the livers of DLY chicks include *AURKA* (-9.10-fold; *p* = 1.50e-30), *BIRC5* (-5.81-fold; *p* = 5.22E-23), *RACGAP1* (-7.34-fold; *p* = 4.17E-28), *PLK1* (−12.60-fold; *p* = 1.66E-46), and *INCENP* (−5.04-fold; *p* = 1.97E-26). Those involved in G2/M DNA Damage Checkpoint Regulation (Figure 10B) include, *TOP2A* (−8.73-fold; *p* = 9.93E-43), *BRCA1* (-6.68-fold; *p* = 9.04E-21), *CCNB2* (−5.88-fold; *p* = 2.22E-31), *CKS2* (−6.49-fold; *p* = 2.30E-06), and *CKS1B* (−8.31-fold; *p* = 1.53E-26). Those involved in Kinetochore Metaphase Signaling (Figure 10C) include, *BUB1B* (−7.25-fold; *p* = 7.87E-27), *KNL1* (-8.57-fold; *p* = 5.08E-20), *CDK1* (−9.11-fold; *p* = 1.58E-37), *CDC20* (−6.71-fold; *p* = 8.39E-28), *SPC25* (−8.63-fold; *p* = 5.47E-35), and *NDC80* (−8.50-fold; *p* = 6.68E-32). Many genes involved in mitosis and apoptosis (Figure 10D, Figures 11, 12) also displayed a greater than 5-fold difference in hepatic expression between DLY and FED chicks.

## 4 Discussion

In our previous work we found that many metabolic genes and their regulators are dynamically expressed in the liver during the embryonic-to-hatch transition and many of these genes are further altered by delaying access to feed for 48 h post-hatch (Hicks et al., 2017; Hicks et al., 2019). These genes are associated with a number of lipid metabolic and carbohydrate metabolic processes. To identify as yet undiscovered molecular mechanisms linked to the hepatic metabolic switch, we used RNA-seq to develop comprehensive hepatic transcriptomes of late-stage (E18) embryonic broilers and peri-hatch (D2) chicks which were either fed-from-hatch (FED) or were not fed (DLY) to retard the metabolic shift. We found 2,430 differently expressed (FC ≥ 2; FDR *p*-value ≤0.05) genes between E18 embryos and FED chicks and 1979 differentially expressed genes between E18 embryos and DLY chicks, with 1,137 genes shared between the E18/FED and E18/DLY comparisons (Figure 1). Between DLY and FED chicks with found 880 differentially expressed genes with 203 genes unique to the DLY/FED comparison and 165 genes shared between all three comparisons (Figure 1). Pathway analyses of these genes suggests that feed consumption after hatch not only alters many metabolic pathways but also has significant impact on a number of pathways related to cell cycle progression, DNA replication and mitosis. Expression patterns suggest that immediate feed consumption after hatch establishes the molecular mechanisms governing proper growth the of liver and delaying access to feed has a significant detrimental impact on these processes, which may negatively impact long-term hepatic function and contribute to growth consequences observed in delayed-fed birds.

As discussed above post-hatching there is a rapid rise in glucose levels due to both increased glucose production from fed intake as well as increased breakdown of hepatic glycogen stores (Noble and Cocchi, 1990). In line with this, Ingenuity Pathway analysis of differentially expressed genes (FC ≥ 2; FDR *p*-value ≤0.05) between FED chicks and E18 embryos predicted an increase (activation z-score 2.112; *p* = 3.95E-15) in “concentration of d-glucose,” with 112 genes differentially expressed. Between DLY chicks and E18 embryos, 84 genes associated with “concentration of d-glucose were differentially expressed (FC ≥ 2; FDR *p*-value ≤0.05), however the resulting z-score (1.681; *p* = 1.56E-10), while positive was not sufficient to determine that this pathway is activated. Additionally, IPA suggests that DLY chicks have higher rates of hepatic fatty acid oxidation than E18 embryos, as “metabolism of acylglycerol” was predicted to have increased activation on DLY chicks with a z-score of 3.126 (*p* = 2.42E-10). As there is an inverse relationship between fatty acid availability and the rate of glucose oxidation (Wolfe, 1998), the potential modest increase in glucose concentration in DLY chicks relative to E18 embryos, may be due a lower availability of fatty acids in DLY chicks from increased utilization, triggering a release of any remaining glucose stores. This further supported by increased circulating glucose levels in DLY chicks compared to E18 embryos (Hicks et al., 2019).

A strong and complex link has been established between the cell cycle and mitotic pathways and lipid metabolism, particularly those involving cholesterol (Fernández et al., 2004). This is of particular importance in the liver. The liver is one of the few tissues which naturally produce polyploidy (containing more than two sets of homologous chromosomes) cells, which is not necessarily the result of a pathological condition (Kreutz et al., 2017). Polyploidy hepatocytes are typically 4n or 8n, but can reach much higher levels of chromosomal accumulation. These hepatocytes can also be mono- or multi-nucleated depending of the mechanism of polyploidization. The number of polyploidy hepatocytes naturally increases with age in vertebrates. Hepatic injury (including resections) and metabolic disorders such as steatosis and obesity also increase hepatocyte polyploidization. Polyploidization can also be the result of the disruption of cholesterol synthesis, which can result in cell cycle arrest and abnormal mitosis (Singh et al., 2013). Polyploidization of hepatocytes has also been linked to the metabolic capacity of the liver (Zhang et al., 2019). CDK1 is a Ser/Thr protein kinase and is involved in the modulation of cell cycle progression and the centrosome cycle. *CDK1* knockout mice have much higher rates of hepatocyte polyploidization compared to wildtype mice (Dewhurst et al., 2020). We found that *CDK1* hepatic expression is reduced -9.11-fold (*p* = 1.58E-37) in DLY chicks compared to FED chicks. *CDK1* expression was −2.53-fold (*p* = 8.54E-11) lower in DLY chicks compared to E18 embryos and was 3.73-fold (*p* = 2.93E-15) higher in FED chicks compared to E18 embryos. Like CDK1, BUB1B is also a Ser/Thr protein kinase. BUB1B is involved in spindle checkpoint regulation to maintain correct chromosome segregation by blocking progression to anaphase (Karess et al., 2013). Disruption of *BUB1B* expression *in vitro*, increased polyploidization of cells and induced mitotic spindle damage and apoptosis (Shin et al., 2003). In DLY chicks *BUB1B* hepatic expression is −7.25-fold lower (*p* = 7.87E-27) than in FED chicks. *BUB1B* expression is 2.37-fold (*p* = 2.74E-08) higher in the livers of FED chicks compared to E18 embryos and is -3.21-fold (*p* = 5.16E-13) lower in DLY chicks compared to E18 embryos. A binding partner of BUB1B is KNL1 (Caldas and DeLuca, 2014). KNL1 acts as a scaffold protein for other kinetochore-associated proteins for proper kinetochore formation. Post-translational modifications that prevent KNL1 interactions induce spindle abnormalities, which results in apoptosis (Caldas and DeLuca, 2014). We found that *KNL1* has a similar expression to other kinetochore-associated genes. *KNL1* is −8.57-fold (*p* = 5.08E-20) lower in the liver of DLY chicks relative to FED chicks, 3.19-fold (*p* = 2.38E-08) higher in FED chicks relative to embryos and -2.75-fold (*p* = 3.11E-06) lower in DLY chicks relative to embryos. Many of the genes associated with kinetochore regulation displayed a similar expression pattern: FED > E18 > DLY. As DLY chicks are forced to continue to use catabolism of yolk cholesterol for energy production and do not have the necessary substrates available for *de novo* cholesterol synthesis, it stands to reason there is less free cholesterol available to use in the production of new cells. An *in vitro* model found that cholesterol starvation increased polyploidization of cells by inhibiting cytokinesis (Fernández et al., 2004). Both NASH patients and NAFLD mice have increased hepatocyte polyploidization compared to their healthy counterparts (Gentric et al., 2015). In the NAFLD mice, this was shown to be the result of inefficient progression through the S/G_2_ phase by activation of the G_2_/M DNA damage checkpoint, preventing the activation of the cyclinB1/CDK1 complex. Activation of the G_2_/M DNA damage checkpoint was one of the major predicted pathways in the DLY/FED transcriptome comparison (Figure 9B). This same study also found that oxidative stress also contributes to increased polyploid hepatocytes (Gentric et al., 2015). Therefore, it is possible that feed consumption after hatch frees up the remaining yolk lipid stores for use in *de novo* hepatocyte production to maintain proper liver growth. Additionally, DLY chicks likely have increased hepatic oxidative stress compared to FED chicks, as their energy must be entirely obtained from oxidation of lipid stores. The transcriptomes generated here suggest that delayed feeding after hatch disrupts the proper progression through the cell cycle and may increase the rate of hepatocyte polyploidization.

Further evidence that delayed feeding after hatch disrupted the innate cell cycle progression and aneuploidy state of the liver is the downregulation of the *FOXM1* regulatory network (Figure 11). FOXM1 is a member of the forkhead family of transcriptional regulators. FOXM1 is expressed by actively dividing cells and is required for completion of the cell division process (Laoukili et al., 2007). FOXM1 inhibition leads to abnormal mitosis, including mis-segregation of chromosomes and cytokinesis failure, which then leads to increased polyploidization (Laoukili et al., 2007). Additionally, inhibition of FOXM1 has been linked to premature cellular aging and its overexpression has been linked to oncogenesis (Laoukili et al., 2007). FOXM1 regulates many cell cycle regulators, particularly those involved in the G2/M phase (Filliol and Schwabe, 2020). During cell cycle progression FOXM1 serves as an activator of: cyclins (including *CCNA2* and *CCNB2*), centromere proteins (including *CENPF*), Ser/Thr proteins kinases (including *PLK1*, *NEK2*, and *AURKA*), and a member of the inhibitor of apoptosis gene family (*BIRC5*) (Filliol and Schwabe, 2020). Other cell regulators governed by FOXM1 include *CDK1* (Chen et al., 2021) and *BUB1B* (Vaz et al., 2021), which, as discussed above are both significantly downregulated in the livers of DLY chicks compared to both E18 embryos and FED chicks. The hepatic expression of numerous members the *FOXM1* regulatory network was also inhibited by delayed feeding (Figure 11). Based on the expression patterns of *FOXM1* network, IPA upstream analysis predicted the activation status of this network to be inhibited with a z-score of −5.184 (*p* = 3.05E-18). Members of this network displayed the same expression pattern; highest hepatic expression in FED chicks, followed by E18 embryos and lowest hepatic expression DLY chicks. *FOXM1* itself had this expression pattern; it was -4.65-fold (*p* = 1.56E-11) lower in DLY chicks relative to FED chicks, -3.04-fold (*p* = 2.09E-11) lower in DLY chicks relative to embryos and +1.66-fold (*p* = 1.21E-02) higher in FED chicks relative to embryos. NEK2 is a Ser/Thr protein kinase which locates to the centromere during the late G2 phase and regulates centromere separation (Fry, 2002). *NEK2* expression was -8.68-fold (*p* = 3.80E-22) lower in the DLY vs. FED comparison, -2.50-fold (*p* = 1.44E-05) lower in the DLY vs. E18 comparison and +3.45-fold (*p* = 1.48E-09) higher in the FED vs. E18 comparison. *AURKA* hepatic expression was −9.10-fold (*p* = 1.50E-30) lower in DLY vs. FED chicks, -2.61-fold (*p* = 2.23E-15) lower in DLY vs. E18 chicks and +3.85-fold (*p* = 4.84E-15) higher in FED vs. E18 chicks. AURKA is associated with spindle pole stabilization as well as centrosome duplication and separation (Asteriti et al., 2015). BIRC5 is involved in the localization of the chromosome passenger protein complex, which serves in chromosome alignment and segregation (Frazzi, 2021). BIRC5 has also been shown to be important in maintaining mitochondrial integrity and function (Hagenbuchner et al., 2013). DLY chicks had −5.81-fold (*p* = 5.22E-23) lower hepatic expression of *BIRC5* than FED chicks and −2.49-fold (*p* = 1.24E-12) lower expression than E18 embryos, while FED chicks had +2.43-fold (*p* = 3.78E-08) higher expression than embryos. Centromere proteins make up part of the kinetochore complex, which associates with the centromere during chromosome separation in mitosis (McKinley and Cheeseman, 2016). Like many other centromere proteins (Figure 10A), *CENPF* expression was significantly downregulated in the livers of DLY chicks compared to FED chicks (−7.99-fold; *p* = 3.13E-23) and compared to E18 embryos (−2.87-fold; *p* = 1.07E-14) and upregulated in FED chicks compared to embryos (+2.88; *p* = 1.63E-10). PLK1 is active during the M phase and plays several essential roles in mitosis, including spindle assembly, mitotic exit and cytokinesis (Asteriti et al., 2015). DLY chicks had -12.60-fold (*p* = 1.66E-16) lower hepatic expression of *PLK1* vs. FED chicks and -3.17-fold (*p* = 1.55E-12) lower expression vs. E18 embryos. FED chicks had +4.24-fold (*p* = 5.50E-17) than embryos. Cyclins interact with cyclin-dependent kinases (CDKs) and function in both their activation and substrate specificity (Johnson and Walker, 1999). CCNA2 interacts with CDK1 and CDK2 to regulate both the G1/S and G2/M transitions (Chotiner et al., 2019). CCNB2 also regulates the G2/M transition *via* interactions with several CDKs, including CDK1 and CDK2 (Li et al., 2019). Hepatic expression of *CCNA2* was -5.45-fold (*p* = 1.40E-25) lower and *CCNB2* was -5.88-fold (*p* = 2.22E-31) lower, respectively, in DLY vs. FED chicks. They were also -2.24-fold (*p* = 5.38 E-E09) and -2.95-fold (*p* = 1.12E-18) lower, respectively, in DLY vs. E18. FED chicks had +2.50-fold (*p* = 1.16E-10) and +2.07-fold (*p* = 1.55E-06) higher *CCNA2* and *CCNB2* expression, respectively, vs. E18 embryos. Taken together, these expression patterns provide additional evidence that part of the metabolic switch involves programming of hepatocyte proliferation activities and delayed feed consumption after hatch may negatively impact liver growth by disrupting mitosis.

The most upregulated gene in both FED and DLY chicks relative to embryos was *CYP7A1*, which encodes cholesterol 7 alpha-hydroxylase. Hepatic *CYP7A1* was 8686.27-fold (*p* = 4.22E-28) higher in FED chicks vs. E18 embryos and 14609.65-fold higher (*p* = 1.63E-33) in DLY chicks vs. E18 embryos. *CYP7A1* was also significantly higher (2.25-fold; *p* = 3.23E-03) in DLY chicks vs. FED chicks. Bile acids are generated in the liver and then secreted into the small intestine (Russell, 2003). Bile acids serve several purposes. First, in the intestine they act as surfactants (i.e. detergents) to enhance absorption of insoluble lipids, including triglycerides and lipolytic products such as fatty acids and 2-monoglycerate (Russell, 2003). Bile acids also serve to excrete excess cholesterol for then liver (Russell, 2003). Excessive bile acids in humans have been shown to reduce both cholesterol biosynthesis and LDL-receptor activity, which in turn, increases plasma levels of LDL-cholesterol (i.e. “bad” cholesterol) (Hofmann, 1999). Excessive buildup of bile acids in hepatocytes has been linked to increased apoptosis *via* mitochondrial damage (Palmeira and Rolo, 2004). A number of apoptotic-associated pathways had increased hepatic transcription in DLY chicks (Figure 12). Taken together, these results provide further evidence that DLY chicks must use their cholesterol stores for energy production rather than cell proliferation, which may contribute to a chronic suboptimal liver functional state, which may explain, in part, the reduced growth rates of DLY birds.

## 5 Conclusion

Over a very short time period, the newly hatched chick must transition from the oxidation of yolk lipids stores (i.e. lipolysis) for the bulk of its energy production to utilizing feed carbohydrates (i.e. lipogenesis and glycolysis) as its main source of energy, in a process called the metabolic switch. Chicks that fail to effectively navigate this switch often have reduced long-term weight gains and reduced growth rates (Julian, 2005). In birds, similar to humans, the liver is the main site of lipogenesis and lipolysis (Hermier, 1997). Over the last several years, we have worked to elucidate the hepatic molecular underpinnings of the metabolic switch. We have discovered that complex transcriptional and post-transcriptional regulatory networks work concordantly to tune metabolic processes to the ever-changing fluctuations in nutrients during the embryo-to-hatch transition. We have also found that these regulatory networks are significantly impacted by delaying feed consumption for 48 h post-hatch. Here, we used RNA-seq to generate unbiased global transcriptional networks in D2 broilers not fed post-hatch. We found that in addition to disturbances in a number of metabolic pathways, unfed chicks had a widespread suppression of gene networks associated with cell proliferation, cell cycle progression and mitosis. The expression patterns of these genes suggests that feed consumption is needed to maintain the rate and integrity of liver growth immediately post-hatch. This may explain, at least partially, why delayed-fed chicks have long-term growth issues. Expression patterns suggest that hepatocytes of delayed-fed birds have abnormal mitosis and increased polyploidization. This may lead to early suboptimal programming of liver growth and function, which then leads to long-term metabolic disturbance. Though additional functional analyses are needed to confirm the mechanisms outlined here, they provide new insight into molecular mechanisms underlying the metabolic switch.

## Data Availability

The datasets presented in this study can be found in online repositories. The names of the repository/repositories and accession number(s) can be found below: https://www.ncbi.nlm.nih.gov/, PRJNA723740.
